# Application of Biostimulants in Tomato Plants (*Solanum lycopersicum*) to Enhance Plant Growth and Salt Stress Tolerance

**DOI:** 10.3390/plants11223082

**Published:** 2022-11-14

**Authors:** Stella Gedeon, Andreas Ioannou, Raffaella Balestrini, Vasileios Fotopoulos, Chrystalla Antoniou

**Affiliations:** 1 Department of Agricultural Sciences, Biotechnology and Food Science, Cyprus University of Technology, Limassol 3036, Cyprus; 2 National Research Council, Institute for Sustainable Plant Protection, 10135 Torino, Italy

**Keywords:** abiotic stress, growth promotion, priming, salinity, tomato

## Abstract

Under the era of climate change, plants are forced to survive under increasingly adverse conditions. Application of biostimulants in plants is shown to mitigate the deleterious effects of abiotic stresses including salinity, enhancing plant tolerance and performance. The present study focuses on the effects of five biostimulants based on biocompost and biofertilizer compounds that have been applied to tomato plants grown in the presence (salt-stressed plants) or absence of salt stress (control plants). To study the beneficial effects of the biostimulants in tomato plants, a series of analyses were performed, including phenotypic and agronomic observations, physiological, biochemical and enzymatic activity measurements, as well as gene expression analysis (RT-qPCR) including genes involved in antioxidant defense (*SlCu/ZnSOD, SlFeSOD, SlCAT1, SlcAPX*), nitrogen (*SlNR, SlNiR, SlGTS1*) and proline metabolism (*p5CS*), potassium transporters (*HKT1.1, HKT1.2*), and stress-inducible TFs (*SlWRKY8, SlWRKY31*). Among all the biostimulant solutions applied to the plants, the composition of 70% biofertilizer and 30% biocompost (Bf70/Bc30) as well as 70% biocompost and 30% biofertilizer (Bc70/Bf30) formulations garnered interest, since the former showed growth promoting features while the latter displayed better defense responses at the time of harvesting compared with the other treatments and controls. Taken together, current findings provide new insight into the beneficial effects of biostimulants, encouraging future field studies to further evaluate the biostimulant effects in plants under a real environment which is compromised by a combination of abiotic and biotic stresses.

## 1. Introduction

Undoubtedly, climate change is putting even more pressure on the resources that feed people and increases the rate of biotic and abiotic stresses on crops, which is raising questions about whether the planet can feed the world or not. Abiotic stresses, such as drought, high salinity, high or low temperatures, hypoxia/anoxia, and nutrient deficiency, affect plant species and often deteriorate crop quality, limiting plant growth and survival [1]. Soil salinity consists of one of the major problems affecting crop productivity, plant growth, fruit quality, and fruit yield in tomato [2]. The tomato (*Solanum lycopersicum* L.) is the second-most important vegetable in the world after the potato, due to its importance in nutrition and health, as well as its high economical value [3]. It is a rich source of many health beneficial compounds such as lycopene, β-carotene, ascorbic acid, vitamin E, and flavonoids, which are correlated with the prevention of certain types of cancer and cardiovascular diseases [4]. Moreover, the tomato contributes to food security, as it is the main ingredient of many products such as soups, juices, purees, and sauces [5]. Because of its importance as food, a lot of research has been done in order to improve tomato crop productivity, fruit quality, and resistance to biotic and abiotic stresses [6].

The negative effects of salinity stress on crops are related to two main factors [7]. The first one is the osmotic stress that occurs by the accumulation of high solute concentrations (Na^+^ and Cl^−^) in the rooting zone. This results in decreased water uptake, in the reduction of stomatal aperture, and consequently affects the transpiration rate. The second factor is ion toxicity due to high ion concentrations in the cytosol (Na^+^ and Cl^−^) [2]. Even though wild tomato genotypes have shown improved salt tolerance in traditional breeding programs, tomato productivity in open field conditions is affected by soil salinity [8], and so alternative strategies are necessary.

Environmental conditions cannot be fully controlled, so it is important to use or even discover diverse strategies on different levels to develop more tolerant cultivars. Humans have been improving plants for thousands of years by manipulating crops using conventional breeding techniques and trying to enhance the genomes of plants towards the desired phenotype, or, more specifically, to make them more tolerant against environmental stresses [9]. In the past decades, the understanding of the molecular basis of plant responses to natural environments or stresses has been the key to genetic engineering. Plant genetic modification methods permit the focused control of specific characters utilizing crop quality and survival [10]. Genome editing tools have proven to be the most innovative and efficient methods for fast and accurate manipulations in crop genomes to protect them against different stresses and improve crop yield [11]. Nevertheless, conventional breeding is a time- and labor-demanding strategy and genetic modification approaches are unacceptable in many countries around the world. Therefore, it is necessary to find some alternative ways, which are also socially acceptable, in order for plants to also successfully tolerate biotic and abiotic stress conditions [12]. 

A promising method of plant tolerance improvement against abiotic stresses is plant priming [13]. Plant priming is achieved by the exogenous application of natural or synthetic priming agents which not only increases tolerance responses of plants but also improves cellular homeostasis and plant growth under stress conditions [12]. A great number of priming agents have been studied to determine their effectiveness against a range of different individually or combined stresses [12,14]. 

Some of the priming agents being evaluated by numerous researchers are biostimulants and/or growth-promoting molecules, and they have been found to affect multiple physiological processes in plants [15]. The application of biostimulants has been considered an innovative agronomic strategy to improve plant growth and productivity and alleviate the negative effects of abiotic stresses [15,16]. While there are many different definitions of biostimulants, the new Regulation (EU) 2019/ 1009 [17] led to the following: “A plant biostimulant shall be an EU fertilizing product the function of which is to stimulate plant nutrition processes independently of the product’s nutrient content with the sole aim of improving one or more of the following characteristics of the plant or the plant rhizosphere: (i) nutrient use efficiency, (ii) tolerance to abiotic stress, (iii) quality traits, or (iv) availability of confined nutrients in the soil or rhizosphere”.

Biostimulants increase plant tolerance to abiotic stress such as drought, extreme temperatures, and salinity, helping plants to recover from the stress damage [18]. The combination of the improved nutrient uptake and enhanced tolerance to stress factors, occurring by biostimulant application, can improve both the quality of output and yielding and thus bring economic benefits especially for the farmers [19]. The most known components considered as biostimulants are mineral elements, vitamins, amino acids, poly- and oligosaccharides, and natural plant hormones. As the years have gone by, several categories of biostimulant products have been made based on their main component or mode of action [18]. The classification referred by Du Jardin (2015) [20] is based on the source of raw material, and it consists of: (1) humic and fulvic acids, (2) protein hydrolysates and other N-containing compounds, (3) seaweed extracts and botanicals, (4) chitosan and other biopolymers, (5) inorganic compounds, (6) beneficial fungi (i.e., mycorrhizal fungi), and (7) beneficial bacteria. The exact mechanisms activated by biostimulants are still being investigated [21]. They can act directly on plant physiology and metabolism or indirectly by improving the absorption of nutrients and other beneficial molecules from the soil (e.g., mycorrhiza) [22] and/or modifying some molecular processes that improve water and nutrient use efficiency in crops [14]. 

The present study aims to examine the stress tolerance and growth promoting the potential of five different, commercially sourced biostimulant solutions, including a biocompost (Bc), a biofertilizer (Bf), their combination in a 70%/30% ratio (Bc70/Bf30 and Bf70/Bc30) and the RNA corrector (named as RNA) which is an advanced version of biocompost solution. “Biocompost solution” is a complete formula that contains nutrients (organic carbon, organic matter, humic and fulvic acids), mineral and trace elements, great quantities of beneficial microorganisms (bacteria, actinobacteria, and fungi), enzymes, and phytohormones. “Biofertilizer solution” is composed of fulvic and humic acids, polysaccharides, carbonates, calcium and inorganic phosphorus, enzymes, and phytohormones. In contrast to the Bc solution, Bf one does not contain any microorganisms. “RNA corrector solution” is a concentrate that contains among other ingredients the active ingredients of the biocompost solution (it is designed to be 50 times more effective/concentrate).

All biostimulants were applied twice in 24-day-old tomato seedlings by watering with a time interval of 7 days between the treatments. After the treatments, half the number of plants were exposed to salt stress, while the other half were kept under control conditions. Morphophysiological evaluation, as well as biochemical and molecular analyses (gene expression of key stress-related genes) were performed to assess the efficiency of the biostimulants as growth promoting and stress ameliorating factors and briefly understand their mode of action. The discovery of novel biostimulants with promising characteristics offers new tools to the farmers to deal with agricultural losses occurring under stress condition. At the same time, the examination of the underlying mechanism responsible for the positive effects of biostimulants application will further enrich our knowledge and open new avenues of a more comprehensive understanding of biostimulants’ *modus operandi*. 

## 2. Results

### 2.1. Phenotypic Observations of Biostimulant-Treated Plants under Control and Salt Stress Conditions

**Figure 1:**
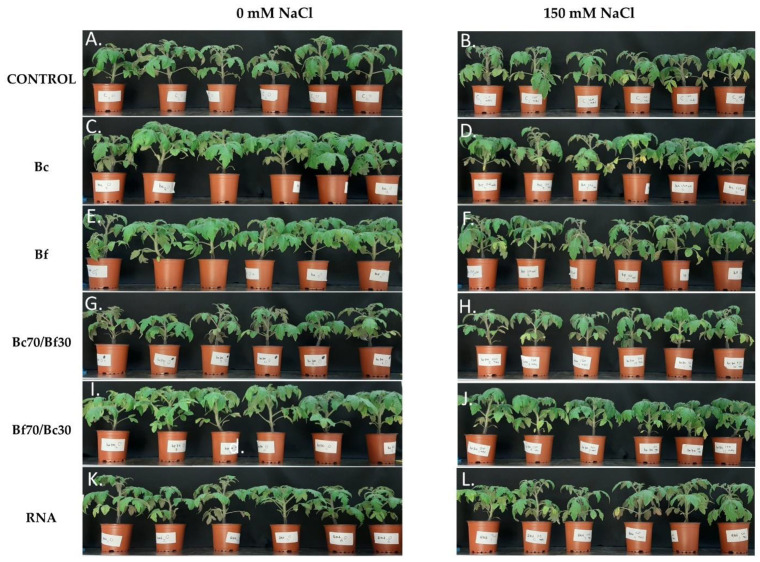
Phenotypic effects of biostimulant application on tomato plants grown in the absence (0 mM NaCl) and presence (150 mM NaCl) of salinity stress with respective controls. (**A**) Control, non-treated and not-stressed, (**B**) Non biostimulant-treated and 150 mM NaCl stressed, (**C**) biocompost (Bc) biostimulant-treated, not stressed, (**D**) biocompost (Bc) biostimulant-treated, 150 mM NaCl stressed, (**E**) biofertilizer (Bf) biostimulant-treated, not stressed, (**F**) biofertilizer (Bf) biostimulant-treated, 150 mM NaCl stressed, (**G**) biocompost 70%/biofertilizer 30% (Bc70/Bf30) biostimulant-treated, not stressed, (**H**) biocompost 70%/biofertilizer 30% (Bc70/Bf30) biostimulant-treated, 150 mM NaCl stressed, (**I**) biofertilizer 70%/biocompost 30% (Bf70/Bc30) biostimulant-treated, not stressed, (**J**) biofertilizer 70%/biocompost 30% (Bf70/Bc30) biostimulant-treated, 150 mM NaCl stressed, (**K**) RNA corrector (RNA) biostimulant-treated, not stressed, (**L**) RNA corrector (RNA) biostimulant-treated, 150 mM NaCl stressed.

The macroscopic observation of plants demonstrated that Bc, Bf, and Bf70/Bc30 biostimulant-treated plants grown under 0 mM NaCl improved growth performance, turgor and greening compared with the respective control (Figure 1A,C,E,I). In addition, the Bc70/Bf30 and RNA corrector-treated plants and non-stressed plants, showed a similar phenotype to control, non-stressed plants (Figure 1G,K,A). The exposure to salt-stress led to intense symptoms of foliar injury, evident as wilting, and chlorotic/necrotic lesions on leaf margins. Bc-treated and salt-stressed plants (Figure 1D) showed extensive wilting and chlorotic symptoms similar to control salt-stressed plants. The other biostimulant-treated plants (Figure 1B,F,H,J,L) had a notably improved phenotype with minimum stress-related damages compared with the respective control plants (Figure 1B). Among all the NaCl stressed-plants, the best growth performance, and the most pronounced mitigating effects to salt stress, were observed in the Bf and Bf70/Bc30 biostimulant-treated plants, in which the turgor was sustained at control non-stress levels, while wilting and necrotic lesions had limited extent (Figure 1F,J, respectively). Taken together, Bf and Bf70/Bc30 biostimulant-treated plants, either exposed to salt stress or grown under control conditions, had the best phenotypic performance, showing optimal growth and mitigation of stress-related symptoms. Moreover, it is worth mentioning that Bc70/Bf30-treated plants presented the most compact structure among all treatments under both salt and control conditions.

### 2.2. Effects of Biostimulants on Growth Parameters

To capture the effects of the sole and joint application of the biostimulants in plant growth, a range of growth parameters were measured at the end of the experiment (Table 1), including (a) plant height, (b) stem width, (c) number of leaves, (d) 3rd leaf fresh weight (fully developed and photosynthetically active leaf), and (e) plant fresh and dry weight. 

First, measurement of growth parameters showed, as expected, that the growth of stressed plants was negatively affected by salinity. Tomato plant height was significantly lower in stressed plants compared with non-stressed plants, except for Bc70/Bf30 and Bf70/Bc30-treated plants. Plants treated with the combination of Bc70/Bf30 were shorter under both stress and non-stress conditions. The number of leaves was not affected by salt stress. Stem width was significantly smaller in stressed plants compared with non-stressed plants, except for control and RNA corrector-treated plants. Bc70/Bf30-treated plants demonstrated less leaves and thinner stem under both stress and non-stress conditions among all biostimulant-treated and control plants. 

Regarding the 3rd leaf FW, it was observed that under both non-stress and stress conditions, Bc70/Bf30-treated plants showed the lower one with respect to the other treatments. Biofertilizer (Bf)-treated stressed plants had a significant reduction in their 3rd leaf FW, while the other treatments had no significant differences between the stressed and non-stressed conditions. As far as plant FW, plants exposed to salt stress demonstrated lower FW compared with the respective non-stress plants. Application of Bf70/Bc30 biostimulant prior to stress imposition significantly increased their FW under both stressed and non-stressed conditions compared with the other biostimulant treatments, while plants treated with Bc70/Bf30 formulation showed decreased FW under both stress and non-stress conditions. DW was significantly increased in Bc and Bf treatments compared with the control plants under non-stressed conditions. Moreover, Bf- and Bc70/Bf30-treated plants weighed less in stressed conditions compared with the non-stressed ones, while there were no significant differences among the other treatments (control, Bc, Bf70/Bc30, and RNA corrector-treated plants) in stressed compared with the non-stressed conditions. Bc70/Bf30 plants weighed less compared with the other treatments under salt-stress conditions. 

The growth capacity of the plants treated with a biostimulant formulation is presented in ratios in Supplementary Table S1. Remarkably, Bc70/Bf30-treated plants demonstrated the most compact structure and the lowest values of all growth parameters. These findings are in line with the ratio analysis, showing that Bc70/Bf30-treated plants to have 8% lower plant height (under both conditions), around 10% fewer leaves (under both conditions), lower FW (22% under non-stress and 27% under salt-stress condition), and 3rd leaf FW (24% under non-stress and 26% under salt-stress condition) and also have shown thinner stem width (11%) and lower DW (27%) under salt-stress condition compared with their respective controls. As far as the other treated plants, Bc-, Bf-, and Bf70/Bc30-treated plants showed increased stem width (16%, 18%, and 23%, respectively), plant FW (30%, 33%, and 47%, respectively), and DW (21%, 25%, and 17%, respectively) under non-stress conditions compared with the non-stressed control plants. On the other hand, the switch combination of Bf70/Bc30 showed the greatest difference in the fresh and dry weight of the plants exposed to stress compared with the respective control plants (25% and 20% increase, respectively). As expected, the results of growth parameters are in agreement with the phenotypic observations.

### 2.3. Physiological Responses of Biostimulants Application

**Figure 2:**
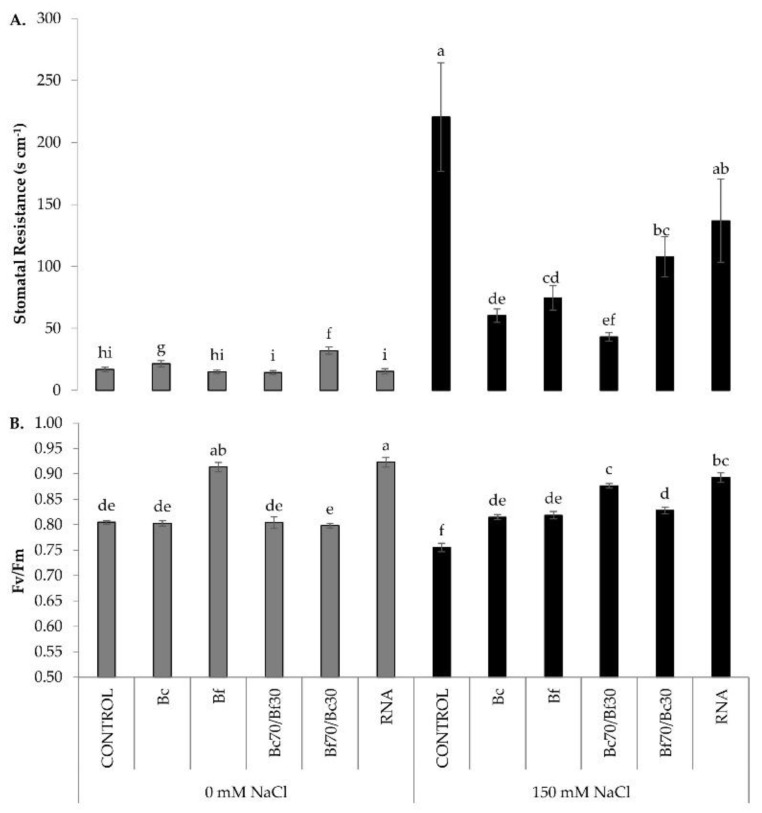
Effect of biostimulant treatments on physiological parameters in control (0 mM) and salt-stressed (150 mM) conditions. (**A**) Stomatal resistance measurement and (**B**) Maximum ^Fv^/_Fm_ photochemical quantum yields of PSII measurement. Data are means ± SE of three replications. Bars with different letters are significantly different (*p* < 0.05). Treatment abbreviations are explained in Figure 1.

The effects of biostimulants in plant stress responses was examined by monitoring the maximum photochemical efficiency of PSII and stomatal resistance, which are two solid indicators of plant physiology performance. Conductivity resistance of the stomata was measured (Rs, s/cm) in fully expanded leaves. Stomatal resistance was similar among plants grown under control conditions with the exception of Bc- and Bf70/Bc30-treated plants, which demonstrated a significantly higher value (Figure 2A). The exposure of plants to salt stress increased the stomatal resistance, with the highest value recorded in non-treated plants, followed by RNA corrector-treated and Bf70/Bc30-treated plants. Conversely, Bc70/Bf30 treatment prior to stress imposition showed the lowest stomatal resistance (i.e., higher stomatal conductance), suggesting that Bc70/Bf30-treated plants are tolerant to salt stress and their capacity to perform gas exchange process is not affected. A similar performance in stomatal resistance was observed in the salt-stressed plants which were pre-treated with the individual biostimulants (Bf, Bc) (Figure 2A). 

Maximum quantum yield of PSII was monitored by measuring leaf chlorophyl fluorescence (^Fv^/_Fm_) using a chlorophyll fluorometer. Measurements of ^Fv^/_Fm_ (Figure 2B) indicated optimum values of 0.8 for all plants growing in the absence of salt stress. Biostimulant pre-treatments did not affect the ^Fv^/_Fm_ ratio in non-stressed plants, except for Bf and RNA treatments which showed significantly higher ^Fv^/_Fm_ ratio. Under salinity, biostimulant-treated plants maintained their fluorescence efficiency at non-stressed levels, except Bf- and RNA corrector-treated plants, which performed better than the non-treated stressed plants despite decreasing ^Fv^/_Fm_ values (Figure 2B). In addition to ^Fv^/_Fm_ measurements, the content of chlorophylls was estimated using a SPAD instrument, with no significant differences recorded among treatments, as shown in Supplementary Figure S1.

### 2.4. Cellular Damage and Osmoprotectant Responses of Biostimulants Treatments

**Figure 3:**
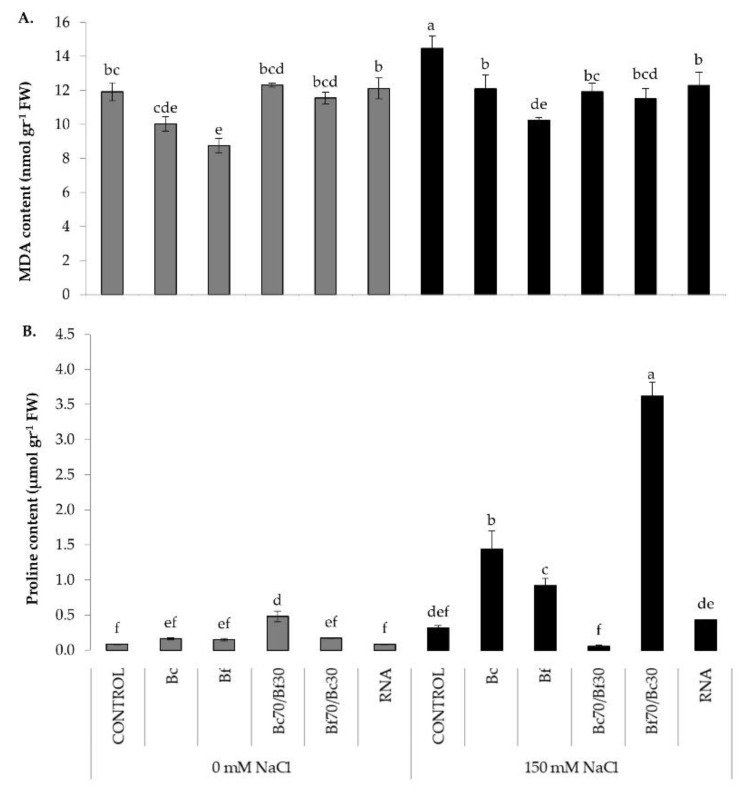
Effect of biostimulant treatments on osmoprotectant and cellular damage indicators production in control (0 mM) and salt-stressed (150 mM) conditions. (**A**) Proline quantification, (**B**) MDA quantification. Data are means ± SE of three replications. Bars with different letters are significantly different (*p* < 0.05). Treatment abbreviations are explained in Figure 1.

Cellular damage was estimated by measuring lipid peroxidation in terms of MDA content. Significant membrane damage was observed under salt conditions, although the pre-treatment with biostimulants showed lower MDA values, providing a statistically significant cellular production in comparison with control salt-stressed plants. Remarkably, plants treated with Bf demonstrated the lowest MDA content both in the present and absence of salt stress. Bf- and Bc-treated plants showed similar MDA content under non-stress conditions (Figure 3A). 

The imposition to salt stress significantly induced the production of the osmoprotectant molecule proline. In non-stressed conditions, Bc70/Bf30 pre-treatment presented the highest value of proline content, while the other treatments did not significantly differ compared with the control. The highest increase in proline content (3.6 μmol/gr) was observed in plants pre-treated with Bf70/Bc30 prior to salinity imposition. A significant but less pronounced increase in proline was recorded in Bc- and Bf-treated plants that were grown under salt conditions (1.4 μmol/gr and 0.9 μmol/gr proline, respectively). On the contrary, Bc70/Bf30 pre-treated plants prior to salt imposition demonstrated the lowest value of proline content. RNA-corrector treatment showed similar levels of proline compared with control NaCl-stressed tomato plants (Figure 3B). 

### 2.5. Regulation of Nitro-Oxidative Homeostasis and Osmoprotectant Biosynthesis on Biostimulant-Treated Tomatoes

**Figure 4:**
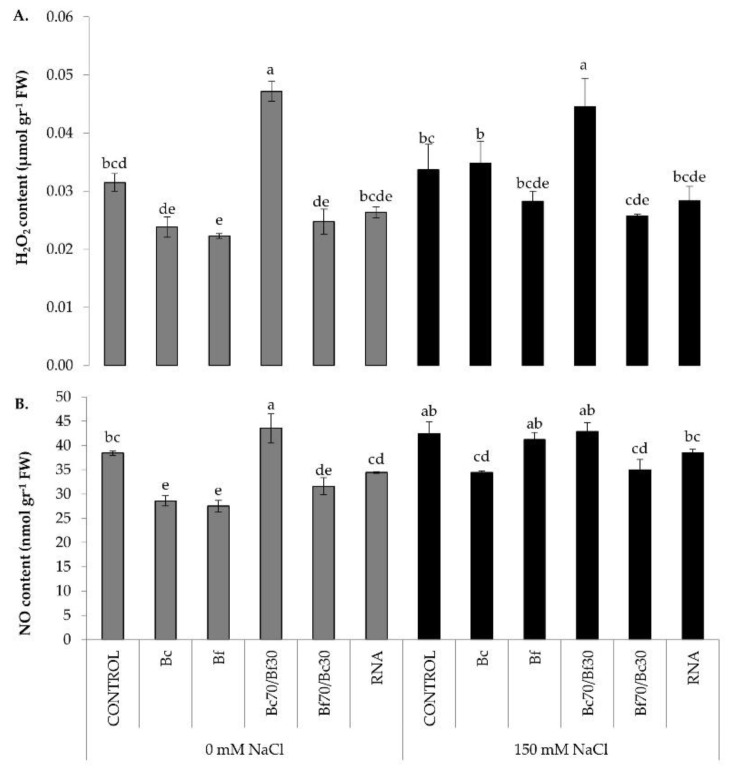
Effect of biostimulant treatments on RONS production, in control (0 mM) and salt-stressed (150 mM) conditions. (**A**) H_2_O_2_ quantification, (**B**) NO quantification. Data are means ± SE of three replications. Bars with different letters are significantly different (*p* < 0.05). Treatment abbreviations are explained in Figure 1.

To evaluate the effect of biostimulant treatments in salt-induced nitro-oxidative stress and whether their application activates the production of RONS independently to stress, H_2_O_2_ and NO were quantified as the major ROS and RNS, respectively. As demonstrated in Figure 4, both reactive species, NO and H_2_O_2_, content followed the same pattern in all treatments and conditions (Figure 4). Bc70/Bf30-treated plants showed the highest NO and H_2_O_2_ content under non-stressed and stressed conditions. The other biostimulants significantly lowered the production of NO and H_2_O_2_ content under non-stress conditions. In turn, Bf70/Bc30- and RNA corrector-treated plants sustained significantly lower levels of H_2_O_2_ and NO compared with non-treated plants under stress conditions (Figure 4A,B). Moreover, Bc- and Bf-pre-treated plants prior to salt imposition had significantly higher RONS content compared with the respective treatments under non-stress condition (Figure 4B).

**Figure 5:**
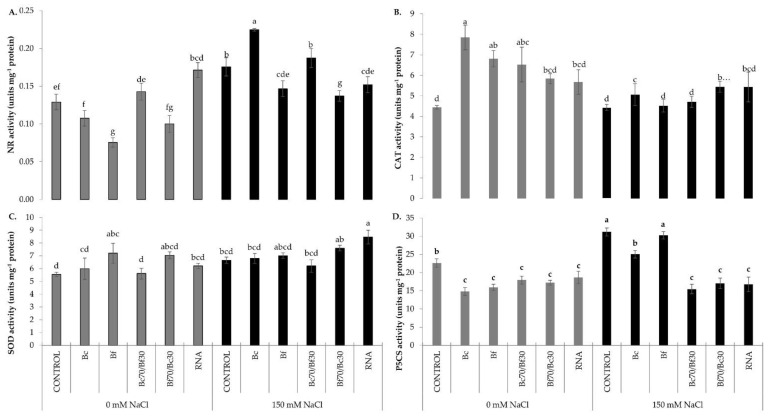
Effect of biostimulant treatments on NR, CAT, SOD, and P5CS enzymatic activity in control (0 mM) and salt-stressed (150 mM) conditions. (**A**) NR enzymatic activity, (**B**) CAT enzymatic activity, (**C**) SOD enzymatic activity, (**D**) P5CS enzymatic activity. Data are means ± SE of three replications. Bars with different letters are significantly different (*p* < 0.05). Treatment abbreviations are explained in Figure 1.

The regulation of nitro-oxidative homeostasis was further studied by measuring the activities of NR which is involved in NO biosynthesis, as well as CAT and SOD which are two key antioxidant enzymes involved in H_2_O_2_ scavenging and generation, respectively. NR activity significantly increased in stressed plants compared with the non-stressed, except for the Bf70/Bc30-treated plants which demonstrated similar levels of NR activity in both stress and non-stress conditions. In salinity conditions, Bc-treated plants had the greatest NR activity which was 22.2% higher than salt-stressed control plants. Contrarily, although Bf70/Bc30 pre-treatment showed the highest NO content, NR activity was 22.2% lower than the activity recorded in salt-stressed control plants. In non-stressed conditions, RNA corrector-treated plants showed significantly higher NR activity, while the Bf treatment demonstrated significantly lower NR activity compared with respective controls. Noteworthy, Bc biostimulant treatment significantly increased NR activity under salt stress by 87.5% compared with the respective treatment on the absence of stress (Figure 5A).

Exogenous application of all biostimulants significantly increased CAT activities compared with control plants under non-stress conditions. The exposure to salt stress significantly decreased CAT activity of biostimulant-treated plants in the levels of control plants, except for Bc treatment. Conversely, SOD activity had no significant differences among the treatments in both non-stress and stress conditions, with some exceptions. Bf-treated plants showed higher SOD activity than the control plants under salt-free conditions, while RNA corrector-treated plants had significantly higher values of SOD activity compared with control plants under salt stress conditions (Figure 5B,C). As for P5CS activity, similar trends in both stress and non-stress conditions were observed, except for the stressed control, Bc- and Bf-treated plants, which had significant higher values than the non-stressed plants. Control plants both under optimum and stress conditions showed the highest P5CS activity values compared with almost all biostimulant treatments (Figure 5D).

### 2.6. Molecular Responses of Biostimulant-Treated Tomato Plants Prior to Salt Exposure 

Relative gene expression analysis was carried out for 14 genes, including the reference gene *Actin*, and the data are presented in Table 2. The studied genes are categorized as followed: (A) antioxidant and other defense genes (*SlCu/Zn-SOD, SlFe-SOD, SlCAT1, SlcAPX*, and *LOX1*), (B) nitrogen and proline metabolism-related genes (*SlNR, SlNiR*, and *SlP5CS*), (C) transporters (*HKT1.1, HKT1.2*, and *SlGTS1*), and (D) transcription factors (*SlWRKY8* and *SlWRKY31*). Fold changes were calculated for each biostimulant treatment using REST-XL analysis and setting the expression levels of 0 mM NaCl as control and salt-stressed (150 mM NaCl) as sample.

The exposure of non-treated, control plants to salt stress significantly downregulated the expression of *NR* and *NiR* genes (fold change, FC were 1.98 and 1.69, respectively), while it revealed significant upregulation of the expression of *Cu/Zn-SOD* gene (FC 4.77). Biocompost-treated, salt-stressed plants significantly upregulated the expression of several genes, including *Fe-SOD, NiR, HKT1.1, WRKY 31*, and *LOX1* with 4.86, 5.22, 2.21, 1.99, and 2.03 FC, respectively. Bf–treated and stressed plants significantly downregulated the genes *Fe-SOD, APX*, and *WRKY* 31 with a FC of 2.50, 1.86, and 1.85, respectively. Bc70%/ Bf 30 %-treated and stressed plants upregulated the expression of *Cu/Zn-SOD, APX, NR, HKT1.2, GTS1, WRKY8*, and *WRKY31* genes (FC, 2.80, 2.80, 2.52, 1.34, 2.26, 2.15, and 2.39, respectively). On the contrary, Bf 70%/ Bc 30%-treated and stressed plants significantly downregulated the expression of *Fe-SOD, CAT, APX, HKT1.2, GTS1*, and *WRKY* 31 genes (fold change 3.18, 2.10, 2.33, 2.83, 2.14, and 2.97 respectively). Lastly, RNA corrector (RNA)-treated stressed plants, upregulated the expression *P5CS, HKT1.1, HKT1.2*, and *LOX1* (fold change 1.91, 1.30, 2.60, and 2.61, respectively). Overall, gene expression analysis demonstrated a differential expression pattern for all examined genes following biostimulant application.

## 3. Discussion

Salinity is one of the major devastating abiotic stresses that significantly reduces crops production, yielding, and productivity [23]. Harmful effects under salinity conditions occur due to the plant’s difficulty in absorbing water from the root environment and its inability to nourish itself [24].These affect the plant at the developmental, physiological, biochemical, and molecular level [25]. In fact, growing tomato plants under salinity stress reduces their yield quantity and quality [26]. These phenomena can be alleviated with the use of biostimulants [27]. Studies have shown that the application of various biostimulants to crop plants makes them more productive, promotes their growth, and improves their tolerance to biotic and abiotic stresses [28,29]. Biostimulant formulations may contain humic acids, fulvic acids, protein hydrolysis products (amino acids), extracts of algae, and beneficial soil microorganisms such as mycorrhizal fungi and plant-growth promoting bacteria [28].

In the present study, five commercially sourced biostimulant solutions were applied to tomato plants, including the biocompost (‘Bc’), the biofertilizer (‘Bf’), their combination in a 70%/30% ratio (‘Bc70/Bf30′ and ‘Bf70/Bc30′), and the RNA (‘RNA corrector’) solution, mainly containing fulvic and humic acids, enzymes, phytohormones, and nutrients, in different proportions in each one. Microorganisms such as bacteria, actinobacteria, and fungi are contained in the biocompost solution, contrarily to the biofertilizer one, which doesn’t contain any. RNA corrector solution, as said above, is a concentrate of biocompost solution. The damaging effects of salt stress were substantially ameliorated by rhizospheric application of almost all biostimulant formulations prior to stress imposition. Moreover, some of the biostimulant applications revealed growth promoting effects in tomato plants grown under non-stress conditions. 

Phenotypic observations showed that optimal growth promotion was achieved in plants treated with Bf and Bf70/Bc30 under both stressed and non-stressed conditions compared with the control and the other treatments. Both contain exogenous polysaccharides that are not present in any of the Bc solutions or in the RNA one. As previously shown, the treatment with exogenous polysaccharides in plants could enhance plant growth, nutrients uptake, and metabolomics profile under non-stressed conditions [30], and induce plant tolerance to salt stress by promoting the antioxidant system and modulating intracellular ion concentration [31]. Moreover, Bf70/Bc30 treatment produced the highest plants, with the thickest stem width. The combination of Bc and Bf solutions contains all the active ingredients and microorganisms of both solutions. It is likely that the presence of microorganisms (Bc) combined with the application of exogenous polysaccharides (Bf) helped plants to better tolerate salt stress. Some microorganisms and more specific rhizobacteria can exude exopolysaccharides to protect themselves and thus their plant hosts against abiotic stresses, including salinity [32]. The application of exogenous polysaccharides enhances plant growth and toleration under stresses [30,31]. Therefore, their combination in the composition of 70% biofertilizer and 30% biocompost (Bf70/Bc30) seemed to be beneficial for the plants, especially under salt stress conditions. Furthermore, both Bf and Bc solutions contain, among others, a mixture of humic and fulvic acids as well as calcium (Ca^2+^). The enhancement in the growth of the tomato plants, after the Bf70/Bc30 treatment, could be attributed to an increased nutrient uptake, as previously described by Türkmen et al. (2004) [33], where humic acids in combination with calcium (Ca^2+^) were used to prime tomato seedlings. Moreover, the application of a tannin-based biostimulant on tomato plants grown under salt stress showed an increase inroot weight and length; a fact that proves the growth promotion effects of biostimulants on tomato [34]. On the contrary, Bf treatment resulted in plants with lower dry weight and 3rd leaf FW under stressed conditions in comparison with the respective treatment under control conditions. In all the other treatments, plants showed the same trends in all growth parameters evaluated in both conditions. A remarkable exception was Bc70/Bf30-treated plants, which showed a compact phenotype owing to lower plant height, number of leaves, dry weight, and 3rd leaf FW in both salt stress conditions compared with the unstressed one. This compact structure possibly improved the capacity of plants to respond faster and adapt in a stressful environment such as salinity. Tomato plants that were inoculated with AM fungi showed a similar compact structure when exposed to water stress [35]. As mentioned before, biocompost (Bc) solution contains a large amount of fungi, among other microorganisms, which may be responsible for the compact phenotype.

Phenotypic observations were further supported by the results of physiological, biochemical, and molecular approaches. Stomatal resistance is the inverse of stomatal conductance that indicates the degree of exchange of CO_2_ and water vapor between environment and inner leaf and has been measured using a porometer. In fact, lower stomatal resistance leads to higher transpiration [36]. The Bc70/Bf30-treated plants, under stressed conditions, showed lower values than the other treatments, though higher than the non-stressed conditions. The other treatments showed higher values in stressed conditions than in the non-stressed ones. Bc and Bf70/Bc30 treatments increased stomatal resistance in the absence of salt stress. Salt-stressed control and RNA corrector-treated plants showed the highest value of stomatal resistance compared with any other treatment. This leads us to the conclusion that these plants closed their stomata to reduce transpiration. As Chaves et al. [37] stated, the defense response of the plants to salinity conditions is the reduction of stomatal conductance and thus the increase of stomatal resistance. However, except for Bc and Bf70/Bc30, the rest of the biostimulant treatments prior to stress imposition demonstrated significantly lower stomatal resistance compared with the control, suggesting the preservation of higher gas exchange rates and transpiration. This is in accordance with a previous study that showed that biostimulant treatments increase stomatal conductivity under saline conditions [38,39]. As an example, the application of glycine betaine significantly increased stomatal conductance of tomato leaves [40].

Biostimulant-treated plants manage to sustain lower stomatal resistance values but higher photochemical efficiency of *PSII* (^Fv^/_Fm_), when grown under salt-stress, enhance further the photosynthetic machinery. This concurs with the findings of Gharbi et al. [41], who found no significant difference in the maximum quantum efficiency of *PSII* (^Fv^/_Fm_) in tomato plants grown under salinity stress. In non-stressed conditions, plants showed equal or higher ^Fv^/_Fm_ values compared with the control plants. Under salinity conditions, all plants treated with a biostimulant formulation had higher ^Fv^/_Fm_ values, which are connected to a better photosynthetic efficiency than the control plants. This shows that biostimulant treatments appear to produce a more efficient *PSII*, giving a better adaption to plant organisms in salinity stress. Peripolli et al. (2021) [42], in measuring the potential quantum yield of photosystem *II* in the pre-morning period in tomato leaves treated with biostimulants under water deficit conditions, found that the application of biostimulants significantly favoured the transport of electrons to photosystem *II* compared with the control treatment, in accordance with our results. 

To further investigate the beneficial effects of biostimulants in plant tolerance to salt stress, cellular damage indicators and the osmoprotectant molecule proline were quantified in non-stressed and salt-stressed plant samples. MDA is a widely used marker of oxidative lipid injury caused by environmental stress. That means that the higher the values of MDA, the more damage has occurred [43]. Among all the treatments in both conditions, Bf had the lowest MDA value followed by Bf70/Bc30-treated plants. This is in accordance with the phenotypic observation in which it was concluded that Bf70/Bc30- and Bf-treated plants had the best visual appearance. Among the other treatments, MDA was significantly increased in control plants under stress conditions compared with non-stress conditions. However, a similar increase was not observed in biostimulant-treated plants. This indicates that possible mode of action of biostimulants is through the protection of cell membrane integrity by minimizing the oxidative stress which lead to lipid peroxidation. This is in accordance with several previous findings, which showed a significant decrease of MDA levels on biostimulants application prior to stress imposition [35,44].

Proline, especially in drought and salinity stress, has an osmoprotectant role and is usually increased under stress conditions, improving the antioxidant system of plants [45]. In this study, it was shown that proline levels also increased under salinity conditions. Under these conditions, Bf70/Bc30 had the highest value of proline content, while Bc70/Bf30 had the lowest one. While Bc70/Bf30 treatment showed the highest proline content in optimum conditions, it showed the lowest one in stressed conditions. Turan et al. (2021) stated that the application of biostimulants decreased proline content under high salinity conditions [44]. In salinity conditions, Bc, Bf, and Bf70/Bc30 treatments had higher proline contents compared with the non-treated and salt-stressed plants. P5CS is a central enzyme that plays a key role in proline biosynthesis. Notably, higher enzyme activity was observed in control plants under both stress and non-stress conditions. Bc- and Bf-treated plants demonstrated higher P5CS activity under salt stress conditions compared with the other treatments, providing biochemical support of the increased proline content found in the respective samples. Contrarily, the expression of *P5CS* was significantly decreased (FC = −3.71) in Bc-treated and salt-stressed plants compared with the respective non-stressed plants (Table 2). This is probably the result of a feedback regulation due to high P5CS enzyme activity and proline production. Alfosea-Simón et al. [46] reported that the foliar application of amino acids (arginine, methionine, glutamine, proline, and tryptophan), either individually or as a mixture, in tomato plants grown with saline water tended to increase the concentration of proline, as the treatments with L-Arg, Met + Arg, and L-Pro had a similar but higher concentration than the rest of the treatments.

The major components of nitro-oxidative responses were also assessed to further decode the role of biostimulants in the alleviation of salt stress detrimental effects. Exogenous application of the formulation Bc70/Bf30 induced the production of H_2_O_2_ under both stressed and un-stressed conditions. H_2_O_2_ is produced predominantly in plant cells during photosynthesis, so an increase in photosynthetic rate may increase H_2_O_2_ production as well. In fact, Foyer and Noctor (2003) [47] described the potentially high capacity of photosynthesis to produce superoxide, hydrogen peroxide, and singlet oxygen, which are buffered by the plants’ antioxidant system. Moreover, Bc70/Bf30 had the lowest proline content in stressed conditions, which may be also associated with the high level of H_2_O_2_. As Gohari et al. [48] recently showed, proline could enhance antioxidant enzymatic activities specifically under salinity conditions to remove H_2_O_2_ and ROS, something that is not happening in this case. The other treatments (with the exception of Bf under stressed conditions) had no significant differences related to stress imposition. Salt stress and RONS production alternates the activity of various important enzymes in plants and usually leads to antioxidant enzyme activation. In general, the first lines of antioxidant defense are, among others, superoxide dismutase (SOD) and catalase (CAT) [49]. CAT activity decreased under salt-stressed conditions in Bc-, Bf-, and Bc70/Bf30-treated plants, while it remained at the same levels in the other treatments. In this case, the low CAT activity levels could be correlated with salt tolerance only because other detoxifying enzymes such as POD, APX, and GR control H_2_O_2_ concentration [50]. Nevertheless, SOD activity had no significant difference between non-stressed and stress conditions. Bano et al. (2012) [51] reported an important reduction of total phenolics, total soluble proteins, and a suppressed activity of catalase, superoxide dismutase, and peroxidase in carrot under saline conditions. 

NO content follows the same pattern as H_2_O_2_ content; while under both the non-stressed and stressed conditions, Bc70/Bf30 had the highest NO content. Previous studies have shown that both NO and H_2_O_2_ function as stress signals in plants, mediating a range of resistance mechanisms in plants under stress conditions [52,53]. Nitrate reductase (NR) catalyses NAD(P)H reduction of nitrate to nitrite and is the key enzyme in the reduction of nitrate (NO_3_^−^) to organic forms within the plant. It is thought to reflect the level of NO production in leaves [54,55]. In stressed conditions, Bc-treated plants followed by Bc70/Bf30 had the greatest values of NR activity. Similarly, the expression of *NR* was increased under salt conditions in Bc70/Bf30 treated plants, while a significant decrease of NR expression was recorded in non-treated salt-stressed plants (Table 2). That comes in accordance with the above results, where Bc70/Bf30 had the highest NO concertation. The fact that NR activity increased under stressed conditions (except in Bf70/Bc30-treated plants) comes in contrast with the fact that salt stress decreases the upregulation, uptake, influx, and NO_3_^-^ reduction, which consequently inhibits NR and NiR activities [56]. Moreover, NR activity was low in some treatments, such as stressed controls and stressed biofertilizer (Bf)-treated plants, while NO contents were high. This may be occurring due to a feedback regulation mechanism that some enzymes are subjected to, with NR enzyme being well known to be regulated by NO itself [57].

As far as RT-qPCR analysis is concerned, fold changes in gene expression of antioxidant related genes, NO and proline biosynthetic genes (*NR*, *NiR*, *P5CS*), transferases, transcription factors, and *LOX* genes among stressed (150 mM NaCl) and non-stressed (0 mM NaCl) conditions were investigated. In this research, upward trends were observed in some biostimulant treatments. Bc-treated and salt-stressed plants which significantly upregulated the expression of antioxidant and NO-biosynthesis related genes (*Fe-SOD, NiR, HKT1.1, WRKY31,* and *LOX1* genes). In addition, Bc70/Bf30-treated plants and salt-stressed plants upregulated the expression of *Cu/Zn-SOD, APX, NR, HKT1.2, GTS1, WRKY 8,* and *WRKY 31* genes, while RNA-treated stressed plants upregulated *P5CS, HKT1.1, HKT1.2,* and *LOX1* genes. These results suggest that the application of Bc in all of its forms, sole (Bc), mixture (Bc70/Bf30), and advanced version (RNA)), activates the molecular mechanisms of plant response to stresses. These results are consistent to those that were previously reported by Ertani et al. (2017) [58]. As currently shown, the application of protein hydrolysate (EM) biostimulants in tomato plants can stimulate plant productivity by activating a series of events such as the activation of TFs (e.g., *WRKY*) that leads to the upregulation of genes involved in defense, antioxidant activities (e.g., *CAT, SOD, APX*), and secondary metabolism. On the contrary, a significant downregulation of *NR* and *NiR* genes and an upregulation of *Cu/Zn-SOD* gene were observed in control salt-stressed plants. Conversely, biofertilizer treatments (Bf and Bf70/Bc30), which do not contain microorganisms, showed a downward trend on gene expression. Bf-treated, stressed plants significantly downregulated *Fe-SOD, APX* genes, and *WRKY 31* TFs. Bf70/Bc30-treated, stressed plants downregulated *Fe-SOD, CAT, APX, HKT1.2, GTS1,* and *WRKY 31* genes. Similar results were reported by Campobenedetto et al. [59], where a lignin-derived biostimulant seed treatment in soybean downregulated genes was involved in stress response, hormone signalling, and primary metabolism, associating the lower activities and lower levels of expression of the corresponding detoxification enzymes with the increased protective effect of this biostimulant. 

To sum up, biocompost-treated (Bc) plants did not display any phenotypic difference with control plants for any of the conditions examined herein (salt stress and non-stressed conditions). Regarding the advanced version of Bc, RNA corrector-treated plants (RNA) showed no significant differences in developmental parameters compared with control plants. Stomatal resistance, proline content, and SOD activity increased in stressed conditions, while *P5CS*, *HKT1.1*, *HKT1.2,* and *LOX1* gene expression was upregulated. Conversely, biofertilizer-treated (Bf) plants had one of the best growth promoting effects under both conditions, also increasing dry and fresh weight compared with the control plants. Furthermore, they showed increased proline content and P5CS activity under stressed conditions, while MDA content was decreased compared with the control plants. Bc treatment downregulated the expression of the antioxidant genes *Fe-SOD* and *APX*. 

Biocompost 70%/Biofertilizer 30 % (Bc70/Bf30) plants had a remarkable compact phenotype (smaller plant size) under both conditions. Moreover, it becomes clear that these plants were affected more by salt-stress (higher H_2_O_2_ and NO content) and activated their detoxifying mechanisms by upregulating a great number of the studied genes, including genes involved in antioxidant machinery (*APX, GTS1, HKT1.2, WRKY8,* and *31*). These findings suggest that Bc70/Bf30-treated plants were able to be more tolerant following stress imposition by keeping a more compact structure (lower and lighter plants with fewer leaves) and spending more energy towards defense. Growth–defense trade-offs are known to occur in plants due to resource restrictions, such as during salt stress. While the deployment of defense mechanisms is imperative for plant survival, defense activation generally comes at the expense of plant growth [60].

On the other hand, application of biofertilizer 70%/biocompost 30% (Bf70/Bc30) demonstrated growth promotion which led to plants with the best growth performance. This is supported by the higher plant height and fresh weight. At the same time, Bf70/Bc30-treated plants had high proline and low MDA, H_2_O_2_, NO content, and NR activity in stressed conditions compared with the stressed controls. Application of Bf70/Bc30 downregulated the expression of many studied genes, including antioxidant genes (*Fe-SOD*, *CAT*, *APX*) and other genes (*HKT1.2*, *GTS1,* and *WRKY 31*). These results suggest that Bf70/Bc30 treatment probably activated the plant antioxidant mechanisms in an earlier stage and then the energy is spent on plant growth and development.

## 4. Materials and Methods

### 4.1. Plant Material and Experimental Treatments 

Tomato (*Solanum lycopersicum* L.) seeds were sown in plastic seedling trays (1 seed per pot) filled with sterile soil, covered with a transparent film, and let to germinate in a growth chamber room under certain conditions of 24/20 °C day/night temperatures, 60–70% RH, with a photosynthetic photon flux density of 120 µmol m^2^ s^−1^ and a 16/8-h photoperiod. At day 8 after sowing, seedlings were transplanted into square plastic pots filled with sterilized pot soil, transferred into a growth room, and let to grow until day 24. Growing plants were watered three times per week. 

Biostimulant solutions used in this study were commercially sourced (De Novo Capital S.ar.l., Rue Alphonse Munchen, Luxemburg). These included three formulations: biocompost, biofertilizer, and RNA corrector solutions and were applied following the manufacturer’s instructions. Biocompost (Bc) is a slightly alkaline solution that contains a high percentage of organic carbon and organic matter, carbonates, humic and fulvic acids with the humic acids being in higher quantities than the fulvic acids. It also contains nitrogen (N) and other mineral elements such as phosphorus (P), potassium (K), calcium (Ca), and magnesium (Mg) and key trace elements such as iron (Fe), manganese (Mn), boron (B), zinc (Zn), copper (Cu), and molybdenum (Mo). On top of mineral and trace elements, biocompost contains a great number of microorganisms such as bacteria, actinobacteria, and fungi and also enzymes and phytohormones of natural origin, such as gibberellins (GA3), auxins (IAA), and cytokinins (ΓΡA). Biofertilizer (Bf) is a basic solution that does not contain any microorganisms. It is composed of fulvic and humic acids with the first one being in greater quantities, polysaccharides, carbonates, calcium (Ca), inorganic phosphorus (P), enzymes, and phytohormones. RNA corrector (RNA) solution is a concentrate that contains among other ingredients the active ingredients of the biocompost solution. It is designed to be 50 times more effective (concentrated) with less volume. Product composition and description are provided in a supplementary file. 

In order to examine the effects of the application of the above biostimulant solutions (and their combinations) in plants under salinity stress, the plants were divided into groups of 12 plants per group and were grouped as follow: CONTROL/0 (untreated plants), CONTROL/150 (plants treated only with 150 mM NaCl), Bc/0 (plants treated only with biocompost (Bc) solution), Bc/150 (plants treated with biocompost solution and 150 mM NaCl), Bf/0 (plants treated only with biofertilizer (Bf) solution), Bf/150 (plants treated with biofertilizer solution and 150 mM NaCl), Bc70/Bf30/0 (plants treated only with 70%Bc + 30%Bf solution), Bc70/Bf30/150 (plants treated with 70%Bc + 30%Bf solution and 150 mM NaCl), Bf70/Bc30/0 (plants treated only with 70%Bf + 30%Bc solution), Bf70/Bc30/150 (plants treated with 70%Bf + 30%Bc solution and 150 mM NaCl), RNA/0 (plants treated only with RNA corrector (RNA) solution), and RNA/150 (plants treated with RNA corrector solution and 150 mM NaCl). 

Plants were treated twice with the biostimulants. At day 24, biostimulants were applied for the first time and each group, except the control group, was root watered by 50 mL of biostimulant solution 2% (Bc, Bf, Bc70/Bf30, Bf70/Bc30, and RNA). Control plants were watered with 50 mL deionized water. After 7 days (day 31), the plants were re-watered with the biostimulant solutions, following the same procedure as the first application. 

Four days after the second application of the biostimulant solutions (day 35), half the plants of each treatment (Bc, Bf, Bc70/Bf30, Bf70/Bc30, and RNA), were treated with 150 mM sodium chloride (NaCl dissolved in water) solution (40 mL), as the other half were watered with deionized water. A second wave of salinity treatment was followed 3 days later (day 38) and applied on the same plants as the previous one. Leaf samples were harvested at day 40 and flash-frozen in liquid nitrogen. All samples stored at −80 °C for subsequent analyses. Experiments were executed in triplicate using pooled material (each replicate consisted of tissue harvested from a minimum of four independent plants). 

### 4.2. Physiological Measurements 

In order to measure the chlorophyll fluorescence emitted from the plants’ leaves, the protocol of the photosynthetic efficiency measurement of photosystem II in a dark-adapted state (^Fv^/_Fm_) was applied, using an OptiSci OS-30p Chlorophyll Fluorometer (Opti-Sciences, Hudson, NH, USA). Leaves had to be dark-adapted for 30 min, before the fluorescence measurements. A ∆T-Porometer AP4 (Delta-T Devices, Cambridge, UK) was used to measure stomatal resistance. Measurements were taken on fully expanded leaves, following the manufacturer’s instructions. Quantification of chlorophyll molecules was achieved, using Chlorophyll Meter SPAD-502Plus and according to the manufacturer’s instructions. 

### 4.3. MDA, Reactive Species and Proline Quantification 

Malondialdehyde (MDA)-thiobarbituric acid reaction was used, as previously described [61], for the MDA content quantification that is commonly used as a lipid peroxidation marker. Leaf tissue hydrogen peroxide (H_2_O_2_) contents were determined spectrophotometrically using potassium iodide (KI) as described by Loreto et al. (2001) [62]. The theory of this method is based on the redox reaction that takes place between H_2_O_2_ and KI. The determination of the nitrogen monoxide (NO) content was achieved using the Griess reagent as Zhou et al. described [63]. Quantification of free proline content had been done by the method described by Bates et al. [63], which is based on the ability of the acid ninhydrin reagent to form a chromogenic complex with proline. The exact proline content was exported from a proline standard curve. 

### 4.4. Antioxidant Enzymatic Activities

The extraction of soluble proteins was achieved by homogenizing leaf samples (100–200 mg) in each protocol iced-cold extraction buffer and after centrifuging each homogenate at 16,000× *g* at 4 °C for 20 min. Every supernatant was collected and used for enzymatic activity and protein content assays. The Bradford method [64] was used for protein quantification using a protein standard curve.

The determination of nitrate reductase (NR) activity, as in NO protocol assay, is based on the diazotization reaction using the Griess reagent and was achieved by following the methodology that Wray and Filner previous described [63]. Total superoxide dismutase (SOD) activity was measured by its ability to catalyze the reduction of nitro blue tetrazolium chloride (NBT) by O_2_^-^ photochemically, as Giannopolitis and Ries reported [63]. Catalase (CAT) activity was determined by the observation of the H_2_O_2_ reduction using the method that was described by Aebi [63]. A more detailed description of the methodology that has been used for SOD and CAT enzymatic activity evaluation can be found in Filippou et al. [63]. The Delta (1)-pyrroline-5-carboxylate synthetase (P5CS) was assayed by measuring the ATP- and NADPH-dependent reduction of glutamate to g-glutamic semialdehyde, and the procedure was carried out following a methodology previously established [65]. All enzymatic activity assay results were expressed as specific activity units per milligram of protein.

### 4.5. RT-qPCR Analysis

Total RNA isolated using NucleoZOL one phase RNA purification, according to the manufacturer’s instruction (Macherey–Nagel, Duren, Germany). The procedure was followed by DNase treatment (RNase-free DNase Set; Qiagen, Hilden, Germany) and RNA precipitation with absolute ethanol and sodium acetate. Nanodrop measurements (Nanodrop 1000 Spectrophotometer, Thermo Scientific, Wilmington, DE, USA) followed to evaluate spectrophotometrically the quality and quantity of extracted RNA. Furthermore, RNA integrity analysis has been done using gel electrophoresis. Reverse transcription method was used for cDNA synthesis, in which 1 µg of total RNA was converted into cDNA using a Primescript 1st Strand Synthesis Kit following the manufacturer’s protocol (Takara, Shiga, Japan). Afterwards, real-time PCR was performed using a Biorad IQ5 thermal cycler (Biorad, Hercules, CA, USA). The reaction mix contained 4 µL cDNA in an RT buffer (diluted 1:5), 0.5 µM of each primer, and 1× master mix (SYBRGreen Super Mix, Invitrogen, San Diego, CA, USA). The thermocycler conditions were set as following: (1) initial denaturation at 95 °C for 2 min, (2) 40 cycles of amplification (95 °C for 5 s, 60 °C for 10 s, and 72 °C for 20 s), (3) melting curve by increasing the temperature per 0.5 °C from 65 °C to 95 °C. A list of the primers used is provided in the Supplementary Table S2. Relative gene expression and statistical analysis (pairwise fixed reallocation randomization test) were performed using REST software according to Pfaffl et al. [66]. Analysis was performed in three biological repeats for each sample and *SlACTIN* [67] was used as a housekeeping reference gene.

### 4.6. Statistical Analysis

Statistical analysis was performed using SPSS v.11 (SPSS Inc., Chicago, IL, USA). One-way ANOVA was initially carried out and then Duncan’s post-hoc pairwise comparison test at *p*-value < 0.05 was used to determine significant differences between individual means, where different letters indicate significantly different values. Data shown represent the mean ± SD.

## 5. Conclusions

Climate change has many consequences in plants, which are forced to survive under extreme environmental stresses, such as increased soil salinity, which greatly inhibits water and nutrient absorption. Biostimulant application in plants is used to improve plant growth and stress tolerance. In the present study, the biostimulant formulations that combined biocompost and biofertilizer showed promising results and therefore a significant potential to enhance plant tolerance when challenged by adverse environmental conditions and/or to induce growth promotion, with noteworthy beneficial effects in agricultural production. Specifically, biofertilizer 70%/biocompost 30% (Bf70/Bc30) and biocompost 70%/biofertilizer 30% (Bc70/Bf30) solutions gained interest, since the former showed the best growth performance and the latter better defense responses at the time of leaf harvesting compared with the other treatments and controls. Remarkably, solo application of Bf enhanced plant growth under both conditions by increasing dry and fresh weight and induced stress tolerance by increasing proline content and P5CS activity as well as decreasing MDA content under salt stress conditions. Conversely, Bc and RNA corrector treatments did not show promising effects on plant growth and stress tolerance.

Future work should focus on translating this approach in other crops of agricultural importance, such as cereals, and evaluate the potential of the most promising biostimulants in field trials, whereby agronomic parameters related to yield and crop quality will be evaluated. This will be further combined with a comprehensive systems biology approach to unravel the exact mode of action in the regulation of plant responses to a stressed environment.

## Figures and Tables

**Table 1:** Effect of biostimulant treatments on plant growth in control (0 mM) and salt-stressed (150 mM) conditions. Plant parameters studied include plant height, number of leaves, stem width, 3rd leaf fresh weight, plant fresh, and dry weight. Data are means ± SE of three replications. Values with different letters in the same column are significantly different (*p* < 0.05). Treatment abbreviations are explained in Figure 1. FW: fresh weight, DW: dry weight.

		Plant Height (cm)		Number of Leaves		Stem Width (cm)		3rd Leaf FW (g)		Plant FW (g)		Plant DW (g)	
		Mean	SE	Mean	SE	Mean	SE	Mean	SE	Mean	SE	Mean	SE
**0 mM *NaCl***	**CONTROL**	14.70^a^	±0.37	8.83^a^	±0.27	0.514^e^	±0.014	2.00^a,b,c^	±0.19	8.71^c,d^	±0.58	1.20^c,d^	±0.07
	**Bc**	14.93^a^	±0.33	8.58^a^	±0.15	0.597^a,b^	±0.011	2.32^a^	±0.15	11.34^b^	±0.80	1.45^a,b^	±0.09
	**Bf**	14.57^a,b^	±0.23	8.50^a^	±0.15	0.605^a,b^	±0.013	2.33^a^	±0.14	11.62^a,b^	±0.47	1.50^a^	±0.07
	**Bc70/Bf30**	13.46^c,d,e^	±0.37	7.83^b,c^	±0.11	0.516^e^	±0.009	1.53^d,e^	±0.07	6.83^e^	±0.22	1.28^b,c,d^	±0.10
	**Bf70/Bc30**	15.18^a^	±0.60	8.75^a^	±0.25	0.633^a^	±0.016	1.90^b,c,d^	±0.16	12.79^a^	±0.41	1.40^a,b,c^	±0.09
	**RNA**	14.80^a^	±0.38	8.58^a^	±0.19	0.534^d,e^	±0.012	1.98^a,b^	±0.12	9.30^c,d^	±0.65	1.31^a,b,c,d^	±0.09
**150 mM *NaCl***	**CONTROL**	13.45^c,d,e^	±0.34	8.33^a,b^	±0.14	0.523^d,e^	±0.009	1.76^b,c,d^	±0.14	8.17^d,e^	±0.46	1.13^d^	±0.05
	**Bc**	13.63^b,c,d^	±0.24	8.75^a^	±0.14	0.558^c,d^	±0.012	2.08^a,b^	±0.10	9.34^c,d^	±0.38	1.23^b,c,d^	±0.04
	**Bf**	12.83^c,d^	±0.31	8.50^a^	±0.15	0.517^e^	±0.012	1.83^b,c,d^	±0.08	8.69^c,d^	±0.41	1.21^c,d^	±0.05
	**Bc70/Bf30**	12.37^d^	±0.27	7.50^c^	±0.15	0.468^f^	±0.017	1.31^e^	±0.05	5.99^f^	±0.32	0.83^e^	±0.03
	**Bf70/Bc30**	14.41^a,b,c^	±0.34	8.67^a^	±0.26	0.577^b,c^	±0.018	1.69^c,d^	±0.10	10.22^b,c^	±0.35	1.35^a,b,c,d^	±0.04
	**RNA**	12.98^c,d^	±0.29	8.25^a,b^	±0.13	0.518^d,e^	±0.011	1.92^a,b,c^	±0.13	8.13^d,e^	±0.41	1.14^d^	±0.05

**Table 2:** Gene expression analysis of selected genes associated with RONS and proline metabolism, enzymatic antioxidants, transporters, lipoxygenase, and salt stress-responsive transcription factors. The numbers represent the fold change (FC) of the genes under salinity (150 mM NaCl) vs. the non-stressed (0 mM NaCl) conditions for each treatment. Bold letters indicate a statistically significant difference at levels *p* < 0.05 = 1 asterisk *, *p* < 0.001 = 2 asterisks **. Treatment abbreviations are explained in Figure 1.

	Fold Change/Treatment	C/150 VS C/0	Bc/150 VS Bc/0	Bf/150 VS Bf/0	Bc70/Bf30/150 VS Bc70/Bf30/0	Bf70/Bc30/150 VS Bf70/Bc30/0	RNA/150 VS RNA/0
	Gene						
**Antioxidant and other defense-related genes**	***SlCu/Zn-SOD***	4.77 **	1.22	−1.78	2.80 *	1.31	1.56
	***SlFe-SOD***	−1.45	4.86 **	−2.50 **	−1.04	−3.18 **	−1.19
	***SlCAT1***	1.06	−1.40	−1.23	−1.20	−2.10 **	1.04
	***SlcAPX***	1.37	−1.34	−1.86 *	2.80 *	−2.33 **	−1.14
	***LOX1***	1.73	2.03 *	−1.96	1.04	−1.09	2.61 *
**Nitrogen and proline metabolism-related genes**	***P5CS***	1.51	−3.71 *	−1.87	−1.02	1.41	1.91 *
	***SlNR***	−1.98 **	2.04	−1.48	2.52 *	1.18	1.07
	***SlNiR***	−1.69 **	5.22 *	−1.20	1.09	−1.06	1.59
**Transporters**	***HKT1.1***	1.69	2.21 *	−1.39	1.50	−1.08	1.30 *
	***HKT1.2***	1.30	1.05	−1.77	1.34 *	−2.83 *	2.60 *
	***SlGTS1***	−1.38	1.26	−1.19	2.26 *	−2.14 *	−1.05
**Transcription factors**	***SlWRKY 8***	1.19	1.24	1.27	2.15 *	−1.68	1.53
	***SlWRKY 31***	1.18	1.99 *	−1.85 *	2.39 *	−2.97 **	1.23

## Data Availability

Not applicable.

## Supplementary Materials

The following supporting information can be downloaded at: https://www.mdpi.com/article/10.3390/plants11223082/s1. Table S1: Ratios between biostimulant-treated plants and their respective control plants in 0 mM NaCl and 150 mM NaCl for all growth parameters studied, including plant height, number of leaves, stem width, 3rd leaf fresh weight, plant fresh, and dry weight. Treatment abbreviations are explained in Figure 1. FW: fresh weight, DW: dry weight. Table S2: *Solanum lycopersicum* primers for RT-qPCR Analysis. * ACTIN used as the reference gene. Figure S1: Effect of biostimulant treatments in chlorophyll content (SPAD) in control (0 mM) and salt-stressed (150 mM) conditions. Data are means ± SE of three replications. Bars with different letters are significantly different (*p* < 0.05). Treatment abbreviations are explained in Figure 1. References are cited in [67,68,69,70,71,72].
