# Supplementary material for: Application of Biostimulants in Tomato Plants (Solanum lycopersicum) to Enhance Plant Growth and Salt Stress Tolerance
Source: Plants (Basel). 2022 Nov 14;11(22):3082. doi: 10.3390/plants11223082 (PMC9693373; doi:10.3390/plants11223082)
Supplement: Supplementary file 1 [file plants-11-03082-s001.zip › Supplementary File 1.pdf]

Properties of the Fertilizer (Liquid and Solid)

1. Improves the physiological condition of the soil with organic matter and improves its porosity. In addition, it improves soils porosity and water absorption
2. It acts as a long-term fertilizer by improving the soil properties in the long term.
3. It is rich in nutritional organic matter with complete and balanced formulation. Rich in nitrogen of organic nature, thus fully absorbable by the plants and more efficient.
4. Unlike other fertilizers, it is a complete formula providing trace elements, substantial for the plants. As a result, the nutritional effect is long term, due to the gradual release of the nutrients depending on the system's (plant-soil) needs.
5. In addition to the nutrients, it contains great quantities of beneficial microorganisms (21 Billion colonies per gr) and enzymes whose action is to activate the natural process of soil rejuvenation and prevent the grow of pathogenic agents. Indeed, due to the microflora presence the pathogens drop to to a safe, non-dangerous level for the plants. Moreover, the abundance of microorganisms stays in the soil even after the cultivating phase and further enriches it, due to the humification of the remaining parts (leaves, roots, branches, etc). That is a secondary fertilizing effect with organic substances.
6. The presence of a sufficient amount of phytohormones of natural origin, such as gibberellins and auxins, is accelerating plant growth regarding time and quantity (leaves, fruit, crop)
7. According to research and in practice, mixing the product with other inorganic fertilizers results in deceleration of the dissolution of their nutrients. (potassium and nitrogen). The result of this effect is the protection of the inorganic fertilizer and the maintenance of its nutritional value for longer time period, due to the gradual release of nutrients.

Biological Analysis (Liquid and Solid)

Phytohormones	Units
Auxins (IAA)	840
Gibberellins (GA3)	270
Cytokinins (ΓΡΑ)	18

Chemical Analysis (Liquid and Solid)

Humidity	40,95%
Dry matter	59,05%
PH	7.03
Electrical conductivity (MS/sm)	2850
Ash	21,60%
Carbonates	0.80%
Organic carbon	27,56%
Organic matter	47,50%
Humic acids	16.75%
Fulvic acids	7,35%
Humic/fulvic ratio	2,28
Total nitrogen	4,80%
Phosphorus	1,70%
Potassium	1,90%
Calcium	0,68%
Magnesium	0,38%
Iron	816,60 ppm
Manganese	212,00 ppm
Copper	12,80 ppm
Boron	28,70 ppm
Zinc	27,30 ppm
Molybdenum	1,35

Microbiological Analysis (Liquid and Solid)

Enzymatic activity

Dehydrogenase	4,10
Phosphatase	88,60
Urease	115,20
Nitrogenase	7,40

Microorganisms (Liquid and Solid)

Total	21000000000
Bacteria	19000000000
Actinobacteria	1500000000
Fungi	500000000
Aerobic nitrogen-fixing bacteria	1200
Ammonia-oxidizing bacteria	18500000000
Nitrifying bacteria	4500000000
Denitrifying bacteria	5000000
Aerobic Cellulolytic bacteria	180000000

Liquid

It contains mainly fulvic acids that are immediately active to the plant, humic acids that ensure beneficial soil condition that are also readily active on the plant active. In addition it contains enzymes and phytohormones that accelare the natural nutritional process of the plants.

Chemical analysis of liquid fertilizer

Humin	1,19%
Humic acids	1,46%
Fulvic acids	9,85%
Fulvic/Humic	6,75%
Solid residue (105^{0}C 24h)	12,5%
Conductivity	0,82 *10^4 μ^9
PH	7,00
Polysacharites	775mg/L
Carbonates	1560mg/L
Ca^{2+}	0,13mg/L
Inorganic phosphorus	10,866m/L
Effective Microorganisms	

Mechanism of Actions of Soil Improver Fertilizer

1. Release from the soil of old and new nutritional substances from old and new fertilizing that become absorbable by the plant
2. Reduction of loss, and accordingly less need for fertilizer
3. Reduction of soil salinity resulting from a prolonged dry period or increased use of fertilizers or even from not optimal water quality. The result is the improvement of the environment surrounding the root system and the rapid absorption of nutrients
4. Conversion of non-absorbable forms of substances of the soil to bio available and absorbable mainly of trace elements. (Trace element deficiencies in plants are rarely caused by soil deficiency but due to the inability of the plant to absorb that particular structure. Fulvic acids that are contained in the fertilizer can release these nutrients, thus preventing deficiencies and minimizing the need for further action.
5. Increase of the quantity of the plant juice and its density. That effect is due to the synergistic action of fulvic acids, enzymes and phytohormones. The result is better condition that is more resistant to pathogens, dry periods or extreme weather conditions (e.g. frost, low temperatures and high temperatures)
6. Precocity, quick maturation of fruit and crops / for fruit trees, olive trees, ornamental plants, etc.
7. Prevention of the “breakage” of fruit (e.g. cherries, grapes). The fruit gets bigger and its preservation time is prolonged (eg kiwi, peach, cherry)
8. Accelerates the maturation of the plant.
9. Improves the overall quality of the fruit
10. Prevents bacteria, fungi, insects and worms (eg caterpillar, louse, mildew, olive fruit fly, woolly aphid)

The soil of any cultivation is not a passive contributor to the success of the cultivation.
The enrichment of the soil with foreign substances, natural or chemical fertilizers that nourish the plants is the minimum we could offer the soil.
The protection of the crop with various pesticides is not enough.
In order to maximize our results, we have to change our previous perceptions and adopt new understanding and practices. Such is the understanding of the natural potential of the soil.
Nature, in order to sustain the right nutrition of all forms of life recycles.
Microorganisms existing in the soil play a major role in the recycling process.
Only the strengthening – not the eradication- of the microorganisms of the soil can gradually reverse the negative consequences of human intervention to the soil.
Man in his pursuit to conquer nature has push it to react. The results are bad food quality, nature devastation, ozone depletion, etc.
In order to reverse these consequences, we have to work with nature and not against it.
In accordance with these principles it is important that we don't kill, destroy these microorganisms, but on the contrary, enrich the soil with more beneficial microorganisms and help them in their valuable work.

That is the answer we and our products are offering. We help the cultivation by enriching the soil with microorganisms and enhance their action.
That is what will safeguard the good nutritional value of the crop, our health, the health of the next generations.

The fertilizer has abundance of bacteria, enzymes and phytohormones.
After a natural biological process (maceration, incubation, distillation) the liquid fertilizer is produced. The liquid product has similar characteristics with the solid.

*For full chemical characteristics and action of both liquid and solid fertilizers as well as the mechanism of action see the report.

General use of the Solid Fertilizer

Intensive agricultures, Greenhouse:

Once a year, at the beginning.

During the basic fertilization 200 liters of biological fertilizer per acre

Arboriculture

Depending on the variety and the age of the tree, 2 to 5 liters in each root under the midday shadow around the tree.

Turf

200 liters per $1000m^2$, topdressing followed by generous amounts of water.

For the preparation of the soil for new cultivation.

200 per $1000m^2$.

Ornamental plants:

During the change of the soil 10% of the fertilizer in volume in common peatland soil and for maintenance 2 to 4 spoonsful, around the plant area, every 2 months.

RNA CORRECTOR LIQUID FERTILIZER

It is a concentrate that contains among other ingredients the active ingredients of the solid fertilizer.
Designed to be 50 times more effective (concentrate) with less volume.
The application becomes EASY FAST and HARMLESS
Mainly contains fulvic acids that act directly on the plant
Humic acids that are active soil impovers and act indirectly
Enzymes and phytohormones are capable of accelerating the plants natural process of nutrition
RNA corrector can be combined with all known chemical fertilizers. It is applied during irrigation in 1:100 solution (beginning of irrigation)

Variety	Dosage per $1000m^2$	Periodicity of application
Tomato	5kg	Every week.1st application on 2nd inflorescence. Last application mid of harvest
Cucumber, zucchini	5kg	Every 15 days. 1st application one month after transplanting. Last application mid of harvest
Watermelon, melon	10kg,5kg	Every 30 days.1st application one month after transplanting. Last application mid of harvest
Pepper, eggplant	5kg	Every 15 days. 1st application in the beginning of bunch closure. Last application mid of harvest
Strawberry	10 kg	Every 20 days. 1st application one month after transplanting. Last application mid of harvest.
kiwi	10kg	February, May, June
Apple, pear, peach, cherry	10kg	Early spring, end of spring, beginning of summer.
Rose	5kg	Every 15 days
carnation	5kg	Every 15 days.
Vine, pomegranate	10 kg	Split in 2 doses, beginning of spring
Asparagus, oregano	20kg	Split in 2 doses, beginning of winter

Chestnut, walnut	10kg	Every 20 days, early spring, late spring, early summer until end of September
Faboideae (bean, okra, peas)	5kg	Every week. 1st application on 2nd inflorescence. Last application mid of harvest
Wheat, corn, all cereal	5kg	Every 15 days in 2 doses, also follar application
Potato, tobacco	5kg	2 times

Properties - Advantages

Accelerates the growth and the productivity of plants and flowers (eg clover, lawn, rose, horticulture, trees (olive trees, fruit trees), ornamental plants and shrubs - prolongation of productivity.

Intense blooming and hold of the fruit (fruit and vegetables, fruit trees, olive trees, flowers, ornamental plants)

Precocity and production increase 15-25%
Activation of the metabolism of flowers, plants, trees, fruit and vegetables and shrubs (eg corn, grain, ornamental, cotton, tobacco, clover, olive, vineyards, fruit trees, pomegranates, horticulture, greenhouses, strawberries, blueberries, lawn)

Enhancement of chlorophyll and photosynthesis process (high energy and productivity, plants get an intense, dark green color during the winter.

Protection during the winter and spring period (precocious growth and produce)

Accelerating the ripening of the fruit
Fighting aphids, cleaning the trees from lice, caterpillars, worms, mildew, cycloconium, woolly aphid, dacus, etc.

Prevents cracking of the fruit (eg cherries, grapes, etc.)

The fruit gets bigger and preservation of fruit is prolonged

Applications General Dosage of RNA Corrector- Fertilizer

10 liters in 1000 liters follar spraying

Vineyards, Trees:
before flowering, at fruit setting and fruit growth until maturation.
Horticultural:
From the germ growth and every 15 to 20 days (especially the periods of stress) until before maturation.
Rice, grain, cotton, legumes:
In the vegetative growth and growth of the fruit, that is 1 time +1 time = 2 times

Particularly:
Plant photosynthetic activity is enhanced
Increased pollen fertility, fruit growing, cell division and plant productivity.
Fruit fall is reduced.
Enhanced absorption of soil nutrients from the plant.
Improves the quality of the produced agricultural products (Brix - nutritional density and nutritional value, taste, color, aroma, etc.)
Strengthens the resistance of the plant and fruits to drought, heat and frost.
The defense of plants in diseases, insects, e.g. thrips, aphids, etc.

Storage:
The product is stored in room temperature, away from sunlight.

Dilution for follar spraying and irrigation
1 liter per 50 liters or per 100 liters for flowers, vegetables
1 liter per 20 liters to 25 liters for fertilizing (irrigation and spraying), for increased resistance, prevention and inhibition of lice, worms, bacteria, fungi, cycloconium, etc
Once in bunch closure and repetition during harvest.
