# Supplementary material for: Application of Biostimulants in Tomato Plants (Solanum lycopersicum) to Enhance Plant Growth and Salt Stress Tolerance
Source: Plants (Basel). 2022 Nov 14;11(22):3082. doi: 10.3390/plants11223082 (PMC9693373; doi:10.3390/plants11223082)
Supplement: Supplementary file 1 [file plants-11-03082-s001.zip › Supplementary File 2.pdf]

Supplementary Table S1. Ratios between biostimulant-treated plants and their respective control plants in 0 mM NaCl and 150 mM NaCl for all growth parameters studied, including plant height, number of leaves, stem width, 3rd leaf fresh weight, plant fresh, and dry weight. Treatment abbreviations are explained in Figure 1. FW: fresh weight, DW: dry weight.

RATIO	Plant height	Number of leaves	Stem width	3rd leaf FW (g)	plant FW (g)	plant DW (g)
**Bc/0 VS C/0**	1.02	0.97	1.16	1.16	1.30	1.21
**Bf/0 VSC/0**	0.99	0.96	1.18	1.16	1.33	1.25
**Bc70/Bf30/0 VS C/0**	0.92	0.89	1.00	0.76	0.78	1.06
**Bf70/Bc30/0 VS C/0**	1.03	0.99	1.23	0.95	1.47	1.17
**RNA/0 VS C/0**	1.01	0.97	1.04	0.99	1.07	1.10
**Bc/150 VS C/150**	1.01	1.05	1.07	1.18	1.14	1.09
**Bf/150 VSC/150**	0.95	1.02	0.99	1.04	1.06	1.07
**Bc70/Bf30/150 VS C/150**	0.92	0.90	0.89	0.74	0.73	0.73
**Bf70/Bc30/150 VS C/150**	1.07	1.04	1.10	0.96	1.25	1.20
**RNA/150 VS C/150**	0.96	0.99	0.99	1.09	1.00	1.01

Supplementary Table S2. *Solanum lycopersicum* primers for RT-qPCR Analysis. *ACTIN used as the reference gene.

Gene	Primer	Nucleotide sequence 5′-3′	Ta (°C)	Reference
SlCu/Zn-SOD	*FW*	GGCCAATCTTTGACCCTTTA	60	1
	RV	AGTCCAGGAGCAAGTCCAGT		
SlFe-SOD	*FW*	TAAATAGAGACTTTGGTTCC	60	1
	RV	TATATTTGCCTCTTAACCCT		
SlCAT1	*FW*	TGATCGCGAGAAGATACCTG	60	1
	RV	CTTCCACGTTCATGGACAAC		
SlcAPX	*FW*	TCTGAATTGGGATTTGCTGA	60	1
	RV	CGTCTAACGTAGCTGCCAAA		
HKT1.1	*FW*	TCTAGCCCAAGAAACTCAAAT	60	2
	RV	CTAATGTTACAACTCCAAGGAATT		
HKT1.2	*FW*	TGAGCTAGGGAATGTAATAAACG	60	2
	RV	AGAGAGAAACTAACGATGAACC		
P5CS	*FW*	TCTTTACAGTGGTCCTCCCC	60	3
	RV	TATACGTTCCCCATGCAGCA		
LOX1	*FW*	TGTCTTTGGGTGGAATTGTGG	60	3
	RV	GGATTGCTCAGTTTCCCTTCC		
SlWRKY 8	*FW*	TAATTCTGCCGGAAAGCCTC	60	4
	RV	ATGCTTATTGCCGGTACTCGA		
SlNR	*FW*	GGTTCATCACTCCCGTACCACTT	60	5
	RV	TCTGCTTCACCATATTCTGCTCT		
SlNii	*FW*	GGATTCATGGGATGCCTGACTAGA	60	5
	RV	TTCTCGTGGAACTGCACCAAAGT		
SlGTS1	*FW*	ACACGGAGAAGGAAGGAAAGGGAT	60	4
	RV	TCCGCGATCATAGAGGTCACAACA		
SlWRKY 31	*FW*	ACAACCTATGAAGGGAAGCACA	60	4
	RV	AGGGTGCTCCCATTTCAGAC		
*ACTIN**	*FW*	GAAATAGCATAAGATGGCAGACG	60	6
	RV	ATACCCACCATCACACCAGTAT		

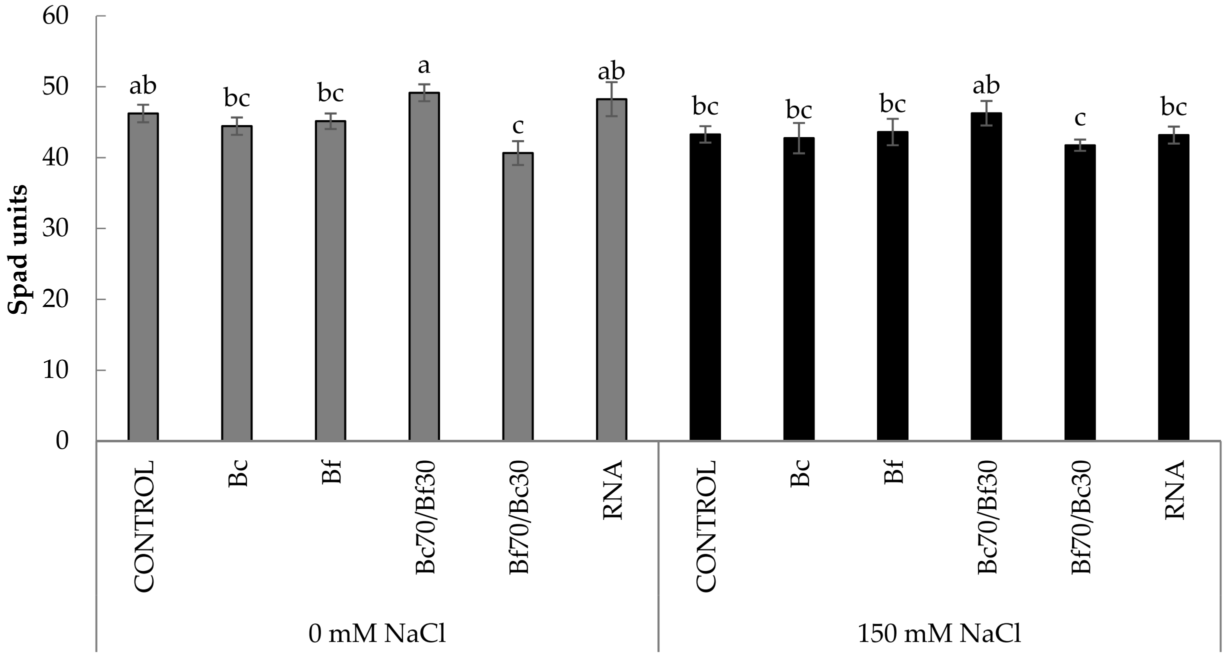

Supplementary Figure S1. Effect of biostimulant treatments in chlorophyll content (SPAD) in control (0 mM) and salt-stressed (150 mM) conditions. Data are means ± SE of three replications. Bars with different letters are significantly different ($p < 0.05$). Treatment abbreviations are explained in Figure 1.

References

1. Martinez, V.; Nieves-Cordones, M.; Lopez-Delacalle, M.; Rodenas, R.; Mestre, T.; Garcia-Sanchez, F.;… Rivero, R. Tolerance to Stress Combination in Tomato Plants: New Insights in the Protective Role of Melatonin. Molecules, 2018, 23(3), 535. doi:10.3390/molecules23030535.
2. Asins, M.J.; Villalta, I.; Aly, M.M.; Olías, R.; Alvarez de Morales, P.; Huertas, R.; Belver, A. Two closely linked tomato HKT coding genes are positional candidates for the major tomato QTL involved in Na+/K+ homeostasis. Plant Cell & Environment, 2013, 36, 1171–1191.
3. Almutairi, Z. Effect of nano-silicon application on the expression of salt tolerance genes in germinating tomato (*Solanum lycopersicum* L.) seedlings under salt stress, 2016, 9. 106-114.
4. Huang, S.; Gao, Y.; Liu, J.; Peng, X.; Niu, X.; Fei, Z.; Cao, S.; Liu, Y. Genome-wide analysis of WRKY transcription factors in *Solanum lycopersicum*. Molecular Genetics and Genomics, 2012, 287,495–513.
5. Lopez-Delacalle, M.; Camejo, D. M.; García-Martí, M.; Nortes, P. A.; Nieves-Cordones, M.; Martínez, V., … Rivero, R. M. Using Tomato Recombinant Lines to Improve Plant Tolerance to Stress Combination Through a More Efficient Nitrogen Metabolism. Frontiers in Plant Science, 2020, 10. doi:10.3389/fpls.2019.01702.
6. Lovdal, T.;Lillo, C. Reference gene selection for quantitative real-time PCR normalization in tomato subjected to nitrogen, cold, and light stress. Analytical Biochemistry, 2009, 387(2), 238–242. doi:10.1016/j.ab.2009.01.024.
